# Artesunate alleviated murine ulcerative colitis by regulating immune response through inhibiting endoplasmic reticulum stress

**DOI:** 10.3389/fimmu.2025.1545468

**Published:** 2025-02-26

**Authors:** Shaojie Yin, Liuhui Li, Xiaolan Chen, Jing Wang, Yujuan Mao, Jingxuan Wang, Xiaoyuting Xie, Jingui Li, Haifeng Yang

**Affiliations:** ^1^ Jiangsu Agri-animal Husbandry Vocational College, Taizhou, China; ^2^ College of Applied Technology, Changzhou University, Changzhou, China; ^3^ College of Veterinary Medicine, Yangzhou University, Yangzhou, China; ^4^ Jiangsu Co-innovation Center for Prevention and Control of Important Animal Infectious Diseases and Zoonoses, Yangzhou, China

**Keywords:** ulcerative colitis, artesunate, endoplasmic reticulum stress, Treg/Th17, macrophage polarization

## Abstract

Innate and adaptive immunity are intricately linked to the pathogenesis of ulcerative colitis (UC), with dysregulation of the Treg/Th17 balance and M2/M1 macrophage polarization identified as critical factors. Artesunate (ARS) has previously been shown to alleviate UC by inhibiting endoplasmic reticulum stress (ERS). To further investigate the regulatory effects of ARS on immune dysregulation associated with colitis and the role of ERS in this process, an experimental colitis model was established using dextran sulfate sodium (DSS). Flow cytometry was employed to assess changes in the Th17/Treg cell ratio in the spleen and macrophage polarization in the intestine, while RT-qPCR was used to quantify the transcription levels of relevant genes in colonic tissues. ARS treatment significantly mitigated DSS-induced pathological damage, reduced the proportion of CD4^+^Th17 cells, and downregulated the mRNA expression of IL-17A, IL-17F, and RORγt, while concurrently increasing the proportion of CD4^+^Treg cells and upregulating TGF-β expression. Additionally, ARS restored the DSS-induced decline in the M2/M1 macrophage ratio and enhanced the transcription of Arg-1 and IL-10, while suppressing the expression of pro-inflammatory markers, including iNOS, IL-1β, IL-6, and TNF-α. Notably, co-treatment with 4-phenylbutyric acid (4-PBA, ERS inhibitor) augmented the immunoregulatory effects of ARS, whereas 2-deoxy-D-glucose (2-DG, ERS agonist) co-treatment counteracted its protective activity against UC. These findings suggest that ERS plays a crucial role in mediating the therapeutic effects of ARS on UC, particularly by modulating Th17/Treg balance and macrophage polarization. This study provides further insights into the mechanistic basis of ARS in UC treatment offering a potential avenue for therapeutic intervention.

## 1 Introduction

Ulcerative colitis (UC) is a chronic, nonspecific inflammatory disorder of the intestine characterized by recurrent episodes of inflammation, typically starting in the rectum and progressively extending into the adjacent mucosa (1, 2). With economic development and improved living standards, the global incidence of UC is gradually increasing. Although plenty of evidence demonstrates that UC is the consequence of the interaction of multiple factors, such as genes, intestinal flora, host immune system, and environment, the pathogenesis of UC has not yet been fully elucidated (3, 4).

In recent years, aberrant activation of both innate and adaptive immunity has been considered a pivotal driver of UC pathogenesis, among which T cells and macrophages play pivotal roles in maintaining intestinal immune homeostasis (5, 6). Th17 cells and Treg cells are two subsets of CD4+ T-effector cells involved in the adaptive immune response. Th17 cells mainly produce IL-17 and exhibit pro-inflammatory effects, while Treg cells play a role in maintaining immune homeostasis, suppressing inflammation, and inducing immune tolerance (7). Research has shown that the Th17 cell ratio increases, while the proportion of Treg cells decreases, in colonic tissues with UC suggesting a Th17/Treg cell imbalance (8). In contrast, restraining the Th17/Treg cell’s lopsidedness is capable of alleviating DSS-induced murine colitis (9).

Macrophages are a type of innate immune cell that participates in almost all immune responses and serves as a bridge between innate immunity and acquired immunity (10–12). In addition, macrophages are plastic cells that reside in various organs and undergo polarized phenotypic changes in response to different tissue microenvironmental stimuli. Generally, macrophages can be divided into M1 classically activated macrophages (pro-inflammatory phenotypes) and M2 alternatively activated macrophages (anti-inflammatory phenotypes) according to their activation state and function (11). Similarly, a decrease in the M2/M1 macrophage ratio is observed in UC, and reprogramming macrophage polarization has been shown to exert a protective effect in experimental colitis (13, 14) indicating that macrophage polarization regulation has the potential to be a novel strategy for UC therapy.

The endoplasmic reticulum (ER) has an extensive endomembrane system linking to other organelles and functions as a sensor for physiological signal variations, adjusting cellular functions, and maintaining intracellular homeostasis (15). Nevertheless, the incorrectly folded proteins in the ER may disrupt the environmental balance, induce ER stress (ERS), and further trigger unfolded protein response (UPR). UPR is a highly conserved and complexly regulated set of pathways that allow cells to respond to stress caused by the accumulation of misfolded and unfolded proteins in the ER (16). Although precise UPR signaling is critical for responding to pathogen infection and tissue stress, constant UPR activation may directly lead to immune tolerance failure, thereby causing the occurrence and development of UC (17). Moreover, UPR plays an essential role in regulating the functions of multiple immune cells. For example, studies suggested that ERS-UPR is involved in the differentiation and effector function of Th17 cells and macrophages in response to inflammatory and autoimmune diseases (18, 19).

Current therapeutic strategies for UC, such as anti-inflammatory drugs and biologics, are still associated with side effects that limit their application and efficacy (20, 21). Hence, there is an urgent need to discover more effective therapeutic drugs with fewer side effects. Artesunate (ARS), well known for its anti-malarial properties, has been shown to possess multiple pharmacological activities, including antimicrobial, antiviral, anti-inflammatory, and antitumor effects (22, 23).

In our previous research, the role of ARS in the UC remission effect was well established, which is mainly associated with the inhibitory effect of NF-κB and the ERS–UPR pathway (24–26). In this context, the DSS-induced experimental murine colitis was established in this study, combined with ERS agonist (2-DG) and inhibitor (4-PBA) to examine the pathological features, the spleen Th17/Treg and colonic M2/M1 cell ratios to further clarify the regulatory efficacy of ARS on immune response, and the role of ERS–UPR in this process.

## 2 Materials and methods

### 2.1 Chemicals and reagents

Artesunate (ARS, A2191, ≥98.5%) was purchased from Tokyo Chemical Industry Co., Ltd. Dextran sodium sulfate (DSS, MW: 36,000–50,000) was purchased from MP Biomedicals (Solon, USA). Viobility 405/520 Fixable Dye (130-109-814), CD11b-VioBright FITC (130-113-243), Anti-F4/80-PE-Vio770 (130-118-459), Anti-Gr-1-APC (130-112-307), CD3-PE-Vio770 (130-116-530), CD4-APC (130-116-526), Anti-IL-17A-PE (130-112-009), Anti-FoxP3-PE (130-111-678) and FcR Blocking Reagent (130-092-575) were all purchased from Miltenyi Biotec (Germany). Brilliant Violet 421 anti-mouse CD86 (105031), PE anti-mouse CD206 (141705), and Brilliant Violet 421 anti-mouse CD25 (102033) were obtained from Biolegend Inc. (USA). FoxP3/Transcription Staining Buffer Set Kit (00-5523-00) was bought from eBioscience Inc. (USA). Cell Fixation and Permeabilization Kit (FMS-FP0100), Brefeldin A (FMS-FZ209), Ionomycin Calcium Salt (FMS-FZ208), and Phorbol 12-myristate 13-acetate (PMA, FMS-FZ207) were purchased from FCMACS (Nanjing, China).

### 2.2 Experimental animals and ethical statement

Female BALB/c mice [20 ± 2 g, SYXK (Su) 2017-0044] were purchased from the Comparative Medicine Centre of Yangzhou University (Jiangsu, China). All mice were reared in specific pathogen-free conditions (22 ± 2°C, 60% humidity) with 12-/12-h light/dark cycle and fed with standard pellet chow and sterile, distilled water *ad libitum*. All animal experiments were conducted according to the guidelines approved by the Animal Care and Use Committee of Yangzhou University.

### 2.3 Experimental colitis induction and treatment protocol

**Figure 1:**
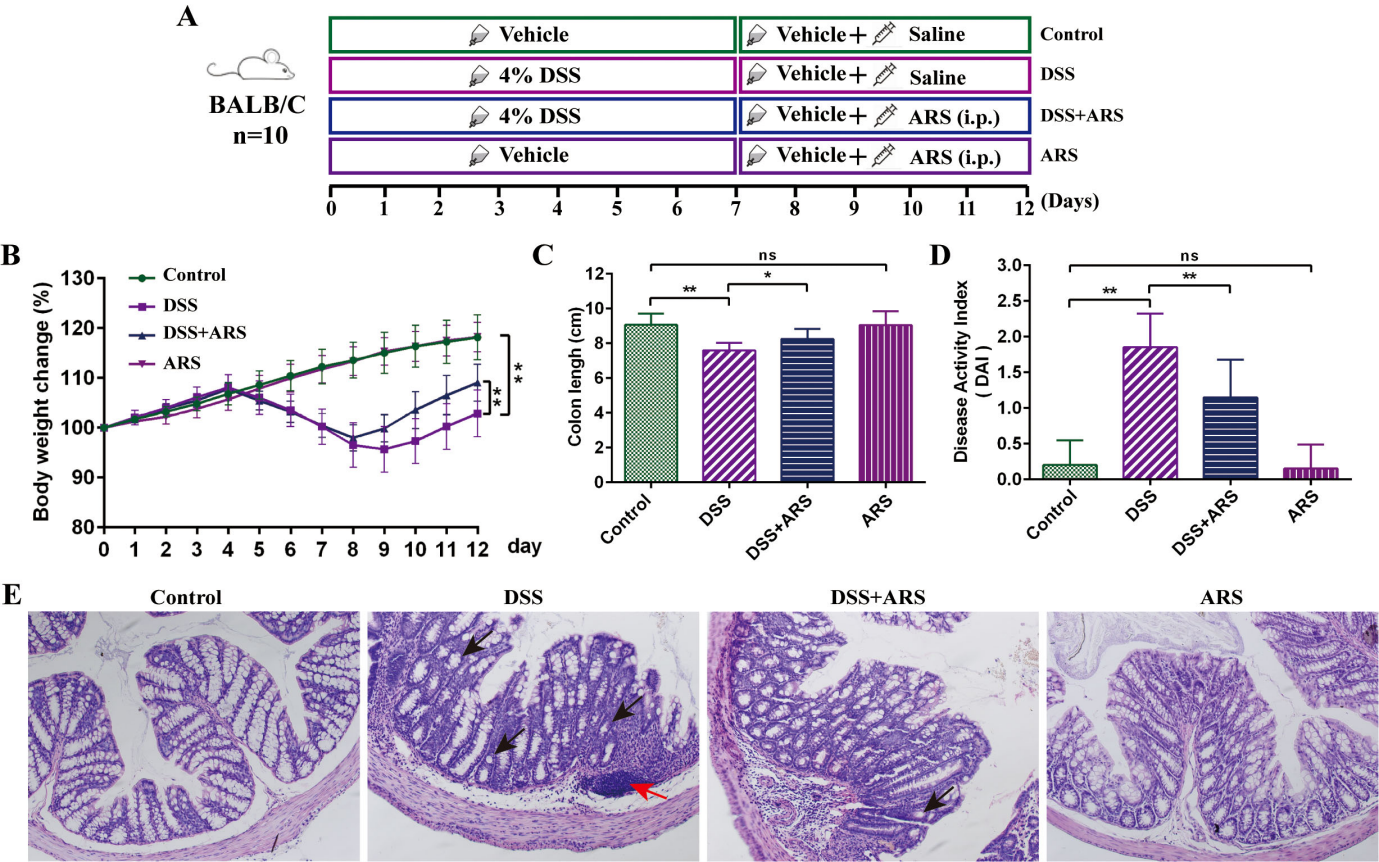
ARS alleviated DSS-induced murine colitis. **(A)** Experimental procedure diagram. **(B)** Body weight gain of mice in each group. **(C)** Colon length analysis of mice in each group. **(D)** Disease activity index (DAI, day 12). **(E)** H&E staining of mouse colon tissue in each group (×200): red arrows indicate inflammatory cell infiltration, and black arrows indicate crypt abnormality and rupture. All data are expressed as mean ± SD. *p < 0.05, **p < 0.01, and ns means no significance.

For the ARS treatment experiment: After a 3-day acclimation feeding, 40 mice were randomly divided into four groups (n = 10) as follows: the Control group, DSS group, DSS + ARS group, and ARS group. In-water administration of 4% (w/v) DSS was applied to establish the experimental colitis model. In addition, mice in the DSS + ARS and ARS groups were lastingly intraperitoneally (i.p.) injected with 30 mg/day/kg of ARS for 5 days after a 7-day DSS consumption (**Figure 1A**).

**Figure 2:**
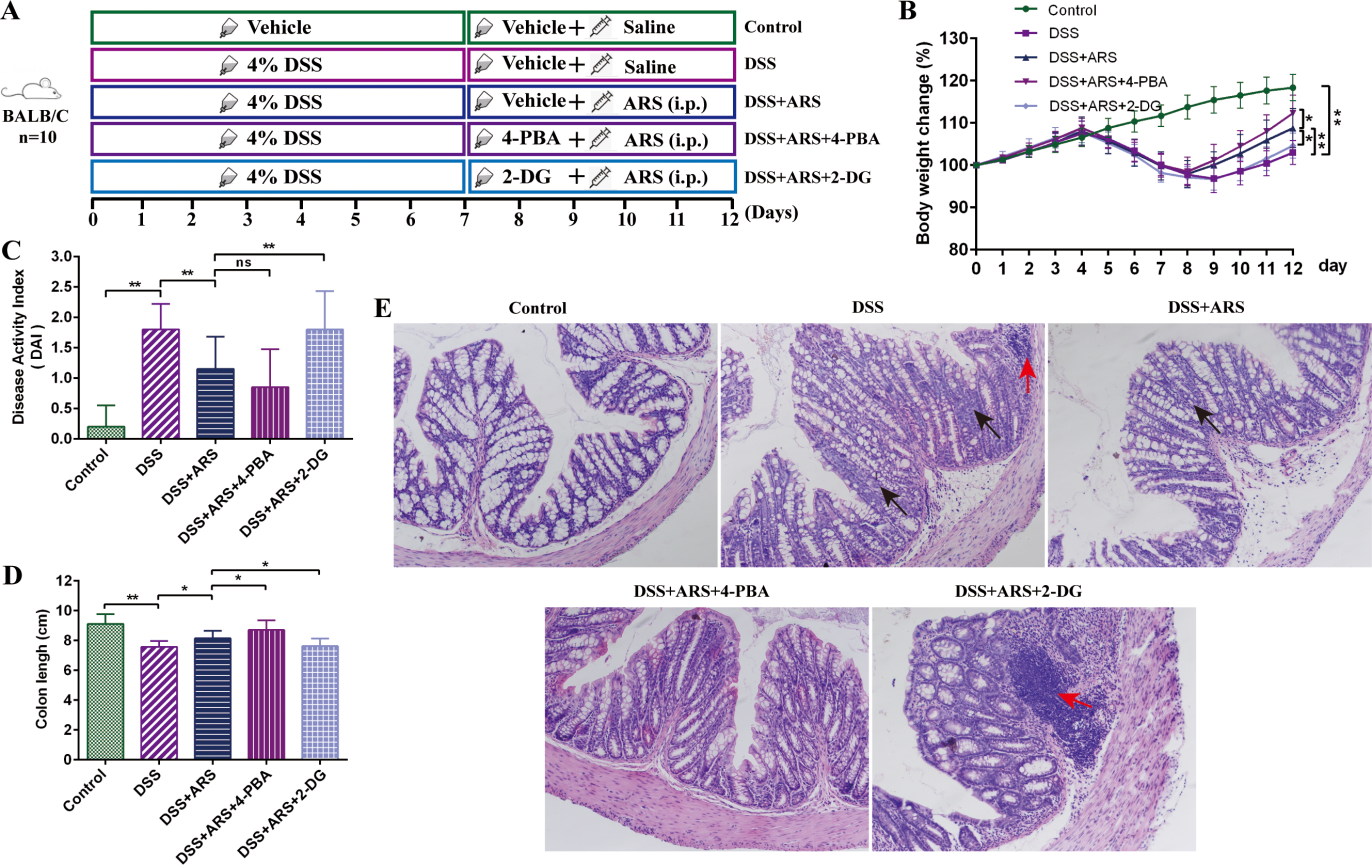
ARS alleviated DSS-induced murine colitis via inhibiting ERS. **(A)** Experimental procedure diagram. **(B)** Body weight gain of mice in each group. **(C)** Disease activity index (DAI, day 12). **(D)** Colon length analysis of mice in each group. **(E)** H&E staining of mouse colon tissue in each group (×200): red arrows indicate inflammatory cell infiltration, and black arrows indicate crypt abnormality and rupture. All data are expressed as mean ± SD. *p < 0.05, **p < 0.01, and ns means no significance.

For evaluating the role of ERS: After a 3-day acclimation feeding, 50 mice were randomly divided into five groups (n = 10) as follows: the Control group, DSS group, DSS + ARS group, DSS + ARS + 4-PBA group, and DSS + ARS + 2-DG group. After inducing colitis with 4% DSS for seven consecutive days, the mice were given distilled water for the following 5 days. Five hundred milligrams per kilogram of 4-PBA and 2-DG were dissolved in drinking water and consumed by the mice through drinking (27). Thirty milligrams per day per kilogram of ARS was inoculated for the ARS intervention (**Figure 2A**).

Body weight and fecal status were monitored daily to calculate the disease activity index (DAI) as previously reported. All mice were sacrificed by cervical dislocation on the last day, and the entire colon tissue was sampled and measured and then fixed in 4% paraformaldehyde or 2.5% glutaraldehyde, or cryopreserved in −80°C for further analysis.

### 2.4 Hematoxylin–eosin staining

After being fixed in 4% paraformaldehyde over 24 h, the distal colon was washed, dehydrated, transparent, and embedded in paraffin wax. Then, the wax was sliced into 4-μm sections and stained with hematoxylin–eosin (H&E) (14).

### 2.5 Single-cell preparation

To prepare the spleen single-cell suspension, the spleens of mice in each group were ground and filtered through a 70-μm nylon-mesh strainer. Red blood cell (RBC) lysis buffer (C3702) was used to lyze RBCs. The cells were washed with precooled PBS two times and diluted into suitable concentrations for further detection.

For colonic single-cell suspension preparation, colonic tissues were repeatedly rinsed with PBS to remove the intestinal contents and cut into small pieces. Then, the mince was separately cultured with 5 mM EDTA to dissociate epithelial cells and digested with 1 mg/ml of collagenase at a 37°C shaker for 30 min. Finally, the colonic single cells were obtained after being washed with precooled PBS and filtered with a 70-μm nylon-mesh strainer.

### 2.6 Flow cytometry assay

Th17 cells: spleen single-cell suspension was incubated with DEME (containing 10% fetal bovine serum, BFA, PMA, and ionomycin) for 7 h in a 37°C incubator, centrifuged at 350 g for 5 min, and the supernatant was removed. Then, CD3–PE–Vio770 and CD4–APC were added into the cell suspension for another 30 min of incubation (shaken every 5 min), centrifuged at 350 g for 5 min, and the supernatant was removed. Cells were then fixed with cell Fixation and Permeabilization A for 15 min and resuspended with Fix and Perm Medium B containing Anti-IL-17A–PE for 15 min, centrifuged at 350 g for 5 min, and the supernatant was removed. Finally, the cells were resuspended in PBS and analyzed by flow cytometry.

Treg cells: spleen single-cell suspension was incubated with CD3–PE–Vio770, CD4–APC, and Brilliant Violet 421 anti-mouse CD25 for 15 min (shaken every 5 min), centrifuged at 350 g for 5 min, and the supernatant was removed. Cells were then resuspended with Foxp3 fixation solvent and cultured for another 30–60 min, centrifuged at 350 g for 5 min, and the supernatant was removed. Then, the cells were suspended with permeabilization solvent containing Anti-FoxP3–PE for 30 min, centrifuged at 350 g for 5 min, and the supernatant was removed. Finally, the cells were resuspended in PBS and analyzed by flow cytometry.

Macrophage polarization: colonic single-cell suspension was incubated with FcR blocking reagent for blocking nonspecific binding sites for 30 min. Then, CD11b–VioBright FITC, Anti-F4/80–PE–Vio770, and Brilliant Violet 421 anti-mouse CD86 were added into the cell suspension for another 15 min of incubation (shaken every 5 min), centrifuged at 350 g for 5 min, and the supernatant was removed. Cells were then fixed with cell Fixation and Permeabilization Kit A for 15 min and resuspended with Fix and Perm Medium B containing PE anti-mouse CD206 for 15 min, centrifuged at 350 g for 5 min, and the supernatant was removed. Finally, the cells were resuspended in PBS and analyzed by flow cytometry.

### 2.7 Real-time-quantitative polymerase chain reaction assay

Total RNA was extracted with Trizol reagents according to the manufacturer’s instructions (R401, Vazyme, Nanjing, China). First-strand cDNA was synthesized according to the HiScript IV RT SuperMix kit (R423, Vazyme, Nanjing, China) after determining the concentration and purity of RNA. Real-time quantitative PCR (RT-qPCR) was performed with a CFX Connect Real-Time PCR System (Bio-Rad, USA) with 2× ChamQ SYBR Green qPCR Master Mix (Q311-02, Vazyme, Nanjing, China). The relative expression of mRNA was calculated by the 2^−ΔΔCt^ method. Additionally, the primers used in this study are listed in **Table 1** and synthesized by Tsingke Biotechnology Co., Ltd., and GAPDH was set as the internal control.

**Table 1:** Primer sequences used for qRT-PCR.

Gene	Primers (5′-3′)	
IL-1β	F R	ATCTCGCAGCAGCACATCA CCAGCAGGTTATCATCATCATCC
IL-6	F R	TTCCATCCAGTTGCCTTCTTG AATTAAGCCTCCGACTTGTGAA
TNF-α	F R	GCAGCCTGTCTCCTTCTATGA TGAAGCAGCAGCCAGCAA
iNOS	F R	GGCTGTCACGGAGATCAATG TGGTAGTAGTAGAATGGAGATAGGA
IL-23A	F R	TAAGAGAAGAAGAGGATGAAGAGAC CTGGAGGAGTTGGCTGAGT
IL-10	F R	AACATACTGCTAACCGACTCCTT GGCAGCCTTGTCCCTTG
IL-4	F R	GCTAGTTGTCATCCTGCTCTTC GGTGTTCTTCGTTGCTGTGA
IL-17A	F R	TGTGTCTCTGATGCTGTTGCT GTGGAACGGTTGAGGTAGTCT
IL-17F	F R	CCTGGAGGATAACACTGTGAGA TTGCTGAATGGCGACGGA
TGF-β	F R	CGCAACAACGCCATCTATGA ACCAAGGTAACGCCAGGAAT
ROR-γt	F R	AAGAGAAGAGGAGAGTGGAACAT GGTGGAGGTGCTGGAAGAT
Arg-1	F R	TGAGAGACCACGGGGACCTG GCACCACACTGACTCTTCCATTC
GAPDH	F R	CACCATCTTCCAGGAGCGAG GGGGCCATCCACAGTCTTC

### 2.8 Statistical analysis

Data were analyzed using a one-way analysis of variance (ANOVA) and an LSD multiple comparison test to determine statistical differences between groups. All statistical analyses were performed using IBM SPSS Statistics software 25.0, and graphs were prepared using GraphPad Prism software 8.0. All experimental data were expressed as mean ± standard deviation (SD) from at least three independent experiments. A value of p < 0.05 was considered a significant difference.

## 3 Results

### 3.1 ARS alleviated DSS-induced murine colitis

As shown in **Figure 1**, a lasting 7-day DSS stimulation significantly induced the classic features, including severe body weight loss, shortened colon, and DAI rising. Concurrently, HE staining showed that the DSS challenge led to colonic crypt and goblet cell abnormality, and inflammatory cell infiltration. Then, the consecutive 5-day ARS intervention alleviated the DSS-caused symptoms and intestinal damage. Besides, ARS alone did not cause any obvious adverse reactions in normal mice (**Figure 1**).

### 3.2 ARS regulated DSS-caused Th17 and Treg cell imbalance

**Figure 3:**
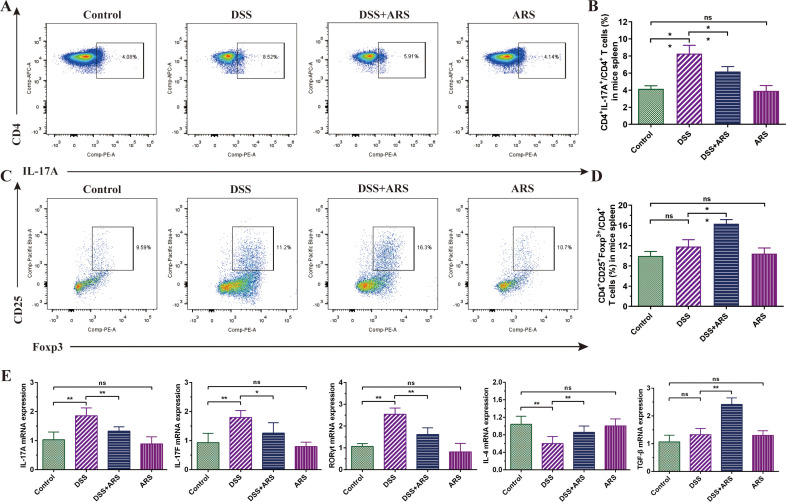
ARS regulated DSS-caused Th17 and Treg cell imbalance. **(A, B)** FCM assay and data analysis of Th17 cells in the spleen. **(C, D)** FCM assay and data analysis of Treg cells in the spleen. **(E)** The relative mRNA expression levels of IL-17A, IL-17F, RORγt, IL-4, and TGF-β in colon tissues were analyzed by RT-qPCR. All data are expressed as mean ± SD. *p < 0.05, **p < 0.01, and ns means no significance.

For evaluating the variation of spleen Th17 cell ratio in CD4^+^T cells, FCM analysis was performed. DSS consumption remarkably increased the number of Th17 cells, while ARS treatment reversed this trend (**Figures 3A, B**). RT-qPCR results showed that ARS significantly inhibited the promotion of RORγt, IL-17A, and IL-17F mRNA expression levels caused by DSS (**Figure 3E**). Meanwhile, DSS increased the ratio of Treg cells in CD4^+^T cells, although there was no significance compared with the Control group. ARS treatment significantly increased the population of Treg cells (**Figures 3C, D**). At the same time, ARS significantly enhanced the transcriptional level of TGF-β and IL-4 mRNA in the colon tissue (**Figure 3E**). Additionally, ARS treatment alone exerted no significant effect on the above indicators (**Figure 3**).

### 3.3 ARS alleviated DSS-induced murine colitis by regulating macrophage polarization

**Figure 4:**
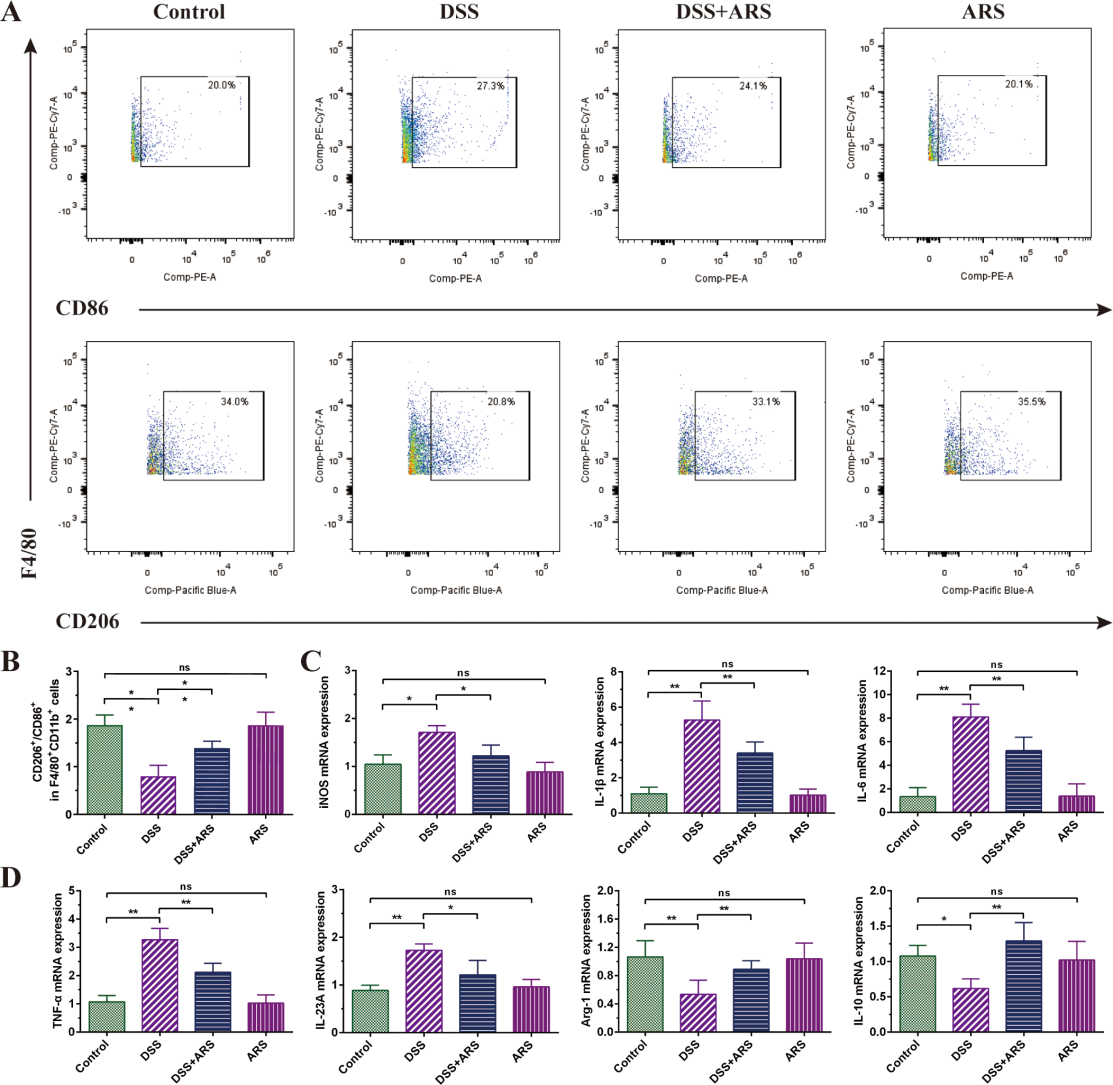
ARS alleviated DSS-induced murine colitis by regulating macrophage polarization. **(A, B)** FCM detection (CD86^+^ and CD206^+^) and data analysis of colonic macrophages. **(C, D)** The relative mRNA expression levels of iNOS, IL-1β, IL-6, IL-23A, TNF-α, Arg-1, and IL-10 in colon tissues were analyzed by RT-qPCR. All data are expressed as mean ± SD. *p < 0.05, **p < 0.01, and ns means no significance.

Compared with the Control group, DSS stimulation significantly reduced the ratio of M2/M1 macrophages in colon tissue, which was restrained by ARS intervention (**Figures 4A, B**). In addition, the DSS challenge upregulated the gene expression level of inflammatory factors IL-1β, IL-6, IL-23A, and TNF-α, as well as the M1 macrophage marker iNOS. Contrarily, ARS treatment significantly inhibited the expression of the abovementioned indicators. Moreover, ARS treatment also noticeably mitigated the inhibitory effect of DSS on the anti-inflammatory factor IL-10 and the M2 macrophage marker Arg-1 mRNA expression level (**Figures 4C, D**).

### 3.4 ARS alleviated DSS-induced murine colitis via inhibiting ERS

The combined use of 4-PBA and 2-DG was applied to further investigate the role of ERS in the remission effect of ARS on DSS-induced colitis. Compared with the single use of ARS, 4-PBA co-treatment further attenuated the DSS-caused body weight and colon length reduction, and decreased DAI scores, along with goblet cell disruption, integrity loss, and inflammatory cell infiltration. In contrast, 2-DG abrogated the remission effect of ARS against UC (**Figure 2**).

### 3.5 ARS regulated DSS-caused Th17 and Treg cell imbalance via inhibiting ERS

**Figure 5:**
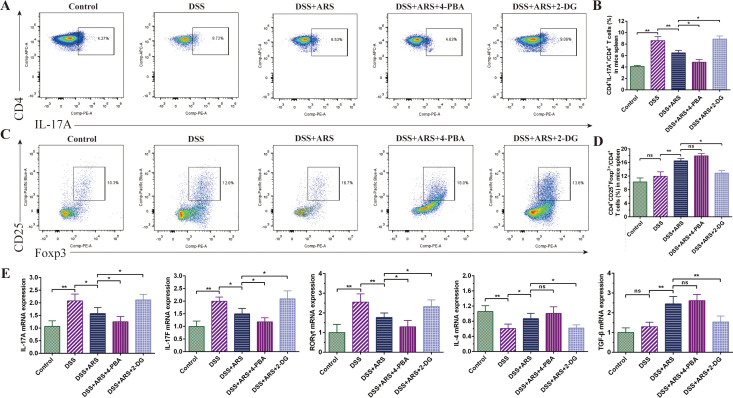
ARS regulated DSS-caused Th17 and Treg cell imbalance via inhibiting ERS. **(A, B)** FCM assay and data analysis of Th17 cells in the spleen. **(C, D)** FCM assay and data analysis of Treg cells in the spleen. **(E)** The relative mRNA expression levels of IL-17A, IL-17F, RORγt, IL-4, and TGF-β in colon tissues were analyzed by RT-qPCR. All data are expressed as mean ± SD. *p < 0.05, **p < 0.01, and ns means no significance.

As shown in **Figure 5A**, ARS treatment significantly inhibited DSS exposure-induced increase in the ratio of Th17 cells in CD4^+^T cells and promoted the expression of RORγt, IL-17A, and IL-17F mRNA in colon tissue. Based on ARS treatment, 4-PBA co-treatment further diminished the ratio of Th17 cells and the mRNA expression level of related cytokines. On the contrary, 2-DG co-treatment momentously compressed the regulatory effect of ARS (**Figures 5A, B, E**).

Congruously, ARS strengthened the Treg cell ratio in the spleen, as well as the expression of TGF-β and IL-4 mRNA. Further analysis revealed that, although 4-PBA co-treatment also extended the ratio of Treg cells and the abovementioned gene expression level, the difference was not significant compared with the ARS-alone treatment group. Concurrently, co-treatment with 2-DG hindered the upgrading regulatory effect of ARS on Treg cells and reversed the promoting effect of ARS on TGF-β and IL-4 transcriptional levels (**Figures 5C–E**).

### 3.6 ARS alleviated DSS-induced murine colitis by regulating macrophage polarization via inhibiting ERS

**Figure 6:**
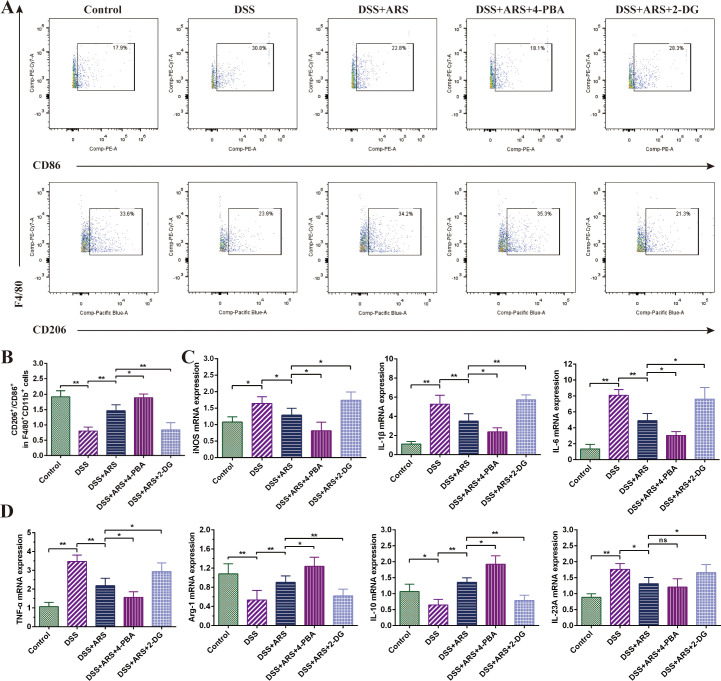
ARS alleviated DSS-induced murine colitis by regulating macrophage polarization via inhibiting ERS. **(A, B)** FCM detection (CD86^+^ and CD206^+^) and data analysis of colonic macrophages. **(C, D)** The relative mRNA expression levels of iNOS, IL-1β, IL-6, IL-23A, TNF-α, Arg-1, and IL-10 in colon tissues were analyzed by RT-qPCR. All data are expressed as mean ± SD. *p < 0.05, **p < 0.01, and ns means no significance.

FCM analysis of spleen cells displayed that ARS medication constrained DSS-triggered colonic M2/M1 macrophage ratio downregulation, accompanied by impeding IL-1β, IL-6, TNF-α, IL-23A, and iNOS mRNA, and promoted the IL-10 and Arg-1 gene expression level. Based on ARS treatment, 4-PBA further escalated the M2/M1 macrophage ratio (**Figures 6A, B**) and mRNA expression of Arg-1 and IL-10 and suppressed the promotion of DSS on the mRNA of inflammatory factors IL-1β, IL6, and TNF-α (**Figures 6C, D**). Contradictorily, 2-DG co-treatment offset the adjusting effect of ARS on the abovementioned indicators.

## 4 Discussion

In the past few years, studies showed that UC onset tends to accompany intestinal immune homeostasis disruption, such as Th17, Treg, and M2/M1 cell imbalance, which further aggravates UC symptoms by increasing the secretion of inflammatory factors and reducing the anti-inflammatory factor expression levels (3, 28). Hence, restoring immune signal balance and remodeling intestinal immune homeostasis could be a novel strategy for UC therapy. In addition, ERS not only induces various cell death processes but also is a key mediator in inducing immune cell dysbiosis (29, 30). Our previous studies have evidenced that ARS significantly inhibited DSS-induced UC by inhibiting ERS through inhibiting the activation of GRP78, PERK–eIF2α–ATF–CHOP, and IRE1α–XBP1s signaling pathway (24). Therefore, this study was conducted to investigate the regulatory efficacy of ARS on DSS-induced immune imbalance and the role of ERS in this process.

Consistent with a previous study (25), DSS consumption induced the hallmark features of UC, including severe weight loss, shortened colon length, increased DAI, and disruption of colonic structure, which were then significantly alleviated by the consecutive 5-day ARS intervention (**Figure 1**). In addition, the combined use of the ERS inhibitor (4-PBA) with ARS remarkably enhanced the remission effect of ARS. Conversely, the agonist (2-DG) further decreased the ARS’s anti-colitis efficacy. These results indicated the excellent therapeutic effect of ARS on UC.

Th17 and Treg cells are both CD4^+^ effector T cells, which play a critical role in the pathogenesis of inflammatory and autoimmune diseases (31). Excessive activation of Th17 cells can lead to intestinal inflammation and intestinal mucosal destruction (32). In contrast, activation of Treg cells, especially CD25^+^Foxp3^+^Treg cells, can inhibit the activation of various immune cells, such as antigen-presenting cells, natural killer cells, and T and B lymphocytes, thereby acting as a highly efficient mediator of peripheral immune tolerance (33). Previous studies have demonstrated that the spleen Th17 and Treg cell ratio can reflect the inflammatory status and outcome of the colon in murine colitis (34). In this study, FCM analysis of spleen Th17 and Treg cells exhibited that continuous exposure to DSS led to a significant increase in the ratio of Th17 cells among CD4^+^T cells. Concurrently, the proportion of Treg cells in CD4^+^T cells showed a slight increase in the DSS groups, but this difference was not statistically significant compared with the control group. In contrast, ARS intervention reduced the ratio of CD4^+^Th17 cells and escalated the CD4^+^Treg cell percentage (**Figure 3**).

RORγt is an essential transcription factor in the development of Th17 cells. TGF-β is a multifunctional cytokine that plays a vital role in regulating T cell-mediated immunosuppression and autoimmunity. Additionally, studies have shown that Th17 cells mobilize, recruit, and activate neutrophils and macrophages by secreting IL-17, mediating local invasion of inflammatory cells and tissue damage, and inducing inflammatory responses, while Treg cells exert anti-inflammatory mechanisms by secreting anti-inflammatory factors such as IL-4 (35, 36). RT-qPCR results manifested that ARS significantly inhibited the RORγt, IL-17A, and IL-17F transcriptional levels in the colon tissue of UC mice and promoted the expression of IL-4 and TGF-β mRNA (**Figure 3**). The above results suggested the therapeutic efficacy of ARS on experimental UC, which is associated with its effect on adjusting DSS-induced Th17/Treg cell imbalance.

As shown in **Figure 2**, based on ARS treatment, 4-PBA co-treatment exerted a more substantial inhibitory effect on DSS-caused Th17 cell increase, along with the mRNA expression level of RORγt, IL-17A, and IL-17F. Contrarily, 2-DG co-treatment significantly curbed the rectified effect of ARS on the above indicators. In addition, although 4-PBA co-treatment did not considerably increase the Treg cell ratio and promote the expression of TGF-β and IL-4 mRNA, 2-DG co-treatment significantly inhibited the positive regulatory effect of ARS on Treg cells and reversed the promotion of ARS on TGF-β and IL-4 gene expression levels (**Figure 5**). The above results revealed that the inhibitory effect of ARS on ERS may be one of the mechanisms by which ARS regulated Th17/Treg homeostasis.

Macrophages are essential to the body’s natural immune system and play a key role in inflammation, host defense, and tissue homeostasis. During the colitis phase, DSS stimulation can induce macrophage infiltration and augment M1 polarization, and M1-polarized macrophages produce excessive inflammatory factors (such as IL-1β, IL-6, and TNF-α), which in turn trigger an inflammatory response and promote the activation of acquired immunity (37, 38). Inversely, M2 exerts a protective effect on the intestinal mucosa by secreting anti-inflammatory factors (such as IL-10), and promoting M2 polarization can effectively alleviate colitis (39). In this study, the DSS challenge significantly decreased M2/M1 macrophage ratio, which was reversed by the ARS treatment. iNOS and Arg-1 are known to be markers of M1 and M2 macrophages, respectively (40). Consistent with the FCM analysis, the results showed that the mRNA expression of iNOS increased, while the mRNA expression of Arg-1 decreased, but ARS treatment counteracted the DSS-induced changes in these two genes. Similarly, ARS also significantly alleviated the promoting effect of DSS on inflammatory factors IL-1β, IL-6, IL-23A, and TNF-α and the inhibitory effect on anti-inflammatory factor IL-10 (**Figure 4**). The above evidence showed that ARS countered DSS-induced colitis by stymying the M1/M2 macrophage ratio.

Research has evidenced that inhibition of the PERK (ERS)-induced signaling pathway can regulate the transition of macrophages from M1 to M2 (41, 42). The 4-PBA co-treatment further enhanced the ARS-induced increase in the M2/M1 ratio, while the 2-DG co-treatment significantly counteracted the regulatory effect of ARS on macrophage polarization. Coherently, the RT-qPCR assay of cytokines’ mRNA expression levels also verified the above consequence (**Figure 6**). The above results suggested that the inhibitory effect of ARS on ERS is involved in the regulation of colonic macrophage polarization and promoted macrophage polarization toward the M2 phenotype, thereby inhibiting inflammation and protecting against colitis in UC mice.

This study confirmed that ERS plays a role in the regulation of ARS on Th17/Treg and M2/M1 balance; however, whether ERS functions as an upstream regulator or a downstream effector in this process remains unclear. Furthermore, the specific molecular targets modulated by ARS have yet to be elucidated. Future research will focus on identifying these targets and delineating the downstream signaling pathways through which ARS mediates ERS and immune regulation.

## 5 Conclusion

To sum up, this study demonstrated that ARS treatment alleviated DSS-induced ulcerative colitis by restoring immune homeostasis. Specifically, ARS inhibited Th17/Treg cell dysbiosis, modulated the M2/M1 macrophage polarization ratio in the colon, and regulated cytokine secretion associated with these immune cells. Furthermore, the involvement of endoplasmic reticulum stress (ERS) in this process was evidenced by the enhanced regulatory effects of ARS upon ERS inhibition and the attenuation of its efficacy when ERS was exacerbated. Combined with the observed improvement in clinical and pathological symptoms, these findings suggest that the modulation of Th17/Treg balance and macrophage polarization via ERS regulation may be a critical mechanism underlying the protective effects of ARS against colitis.

## Data Availability

The raw data supporting the conclusions of this article will be made available by the authors, without undue reservation.
